# ICU scoring systems: which one to use in patients with sepsis?

**DOI:** 10.1186/cc10383

**Published:** 2011-10-27

**Authors:** D Juneja, O Singh, P Nasa, Y Javeri, M Mathur, R Dang

**Affiliations:** 1 Department of Critical Care Medicine, Max Super Speciality Hospital, Saket, New Delhi, India

## Introduction

Disease-severity scoring systems have been developed for stratification of ICU patients. These systems have been tested and validated in various general medical and surgical ICU patients. However, the validity and efficacy of these systems, especially the newer generation, has not been assessed in patients with sepsis, which is the commonest indication for admission to a medical ICU. Hence, we conducted this study to assess the performance of various ICU scoring systems - Acute Physiology and Chronic Health Evaluation (APACHE) II, III, IV; Simplified Acute Physiology Score (SAPS) II, III; Mortality Prediction Model (MPM) II_0_, III_0_; and Sequential Organ Failure Assessment (SOFA) scores - in septic patients admitted to a medical ICU.

## Methods

A prospective, observational study was conducted in a tertiary care medical ICU and consecutive patients fulfilling the diagnostic criteria for sepsis during the first 24 hours of ICU admission were included over a 2-year period. Data related to patient demographics and that required to compute various scores were recorded. Predicted mortality was calculated using original regression formulas. The standardized mortality ratio (SMR) was computed for mortality prediction. Calibration was assessed by calculating the Lemeshow-Hosmer goodness-of-fit C-statistic. Discrimination was assessed by calculating the area under the receiver operating characteristic (AUROC) curves. ICU mortality was the primary outcome measure.

## Results

Data were analyzed for 438 septic patients. The mean age of patients was 64.5 ± 16.3 years and 301 (68.7%) were male. The mean ICU and hospital length of stay was 6.39 ± 9.7 and 9.99 ± 10.5 days, respectively. The observed ICU mortality was 107/438 (24.4%). Mortality predicted by SAPS III score was closest to that of actual mortality with a SMR of 0.98 followed by that of MPM III_0 _(SMR - 1.13) and APACHE IV (SMR - 1.18) scores (Table 1). APACHE IV (χ^2 ^= 4.416; *P *= 0.818) had the best calibration followed by SAPS II (χ^2 ^= 6.073; *P *= 0.639) and SAPS III scores (χ^2 ^= 6.538; *P *= 0.587). There was no statistically significant difference between the AUROCs of these scores; SOFA (AUROC = 0. 0.889) performed the best followed closely by APACHE IV (AUROC = 0.882) and APACHE III (AUROC = 0.880) scores (Table 2).

**Table 1:** Comparison of the actual and predicted mortality rates for the various scoring systems

Variable	Actual mortality	Predicted mortality	SMR
APACHE II	0.244	0.296	0.824
APACHE IV	0.244	0.206	1.18
SAPS II	0.244	0.297	0.822
SAPS III	0.244	0.249	0.98
MPM II_0_	0.244	0.314	0.777
MPM III_0_	0.244	0.216	1.13

**Table 2:** Area under the curve for predicting ICU mortality for various scoring systems

Scoring system	AUC	95% CI	Cut-off	Sensitivity (%)	Specificity (%)
APACHE II	0.880	0.845 to 0.914	>18.5	86.9	75.8
APACHE III	0.880	0.847 to 0.914	>63.5	82.2	70.4
APACHE IV	0.882	0.848 to 0.916	>17.7	83.2	74
SAPS II	0.849	0.808 to 0.890	>41.5	82.2	70.4
SAPS III	0.873	0.838 to 0.907	>53.5	85	72.2
MPM II_0_	0.849	0.807 to 0.891	>30.1	77.6	76.1
MPM III_0_	0.872	0.835 to 0.909	>18.7	81.3	77.9
SOFA	0.889	0.857 to 0.922	>5.5	86.9	79.2

## Conclusion

Overall, the newer generation of scoring systems performed better than their older counterparts and was more accurate. Older scoring systems had a tendency to overpredict mortality. However, all the scores tested had good efficacy and the difference in efficacy was not statistically significant.

